# Knots cascade detected by a monotonically decreasing sequence of values

**DOI:** 10.1038/srep24118

**Published:** 2016-04-07

**Authors:** Xin Liu, Renzo L. Ricca

**Affiliations:** 1 Beijing-Dublin International College & Institute of Theoretical Physics, Beijing University of Technology, 100 Pingleyuan, Beijing 100124, P.R. China; 2 Department of Mathematics & Applications, University of Milano-Bicocca, Via Cozzi 55, 20125 Milano, Italy

## Abstract

Due to reconnection or recombination of neighboring strands superfluid vortex knots and DNA plasmid torus knots and links are found to undergo an almost identical cascade process, that tend to reduce topological complexity by stepwise unlinking. Here, by using the HOMFLYPT polynomial recently introduced for fluid knots, we prove that under the assumption that topological complexity decreases by stepwise unlinking this cascade process follows a path detected by a unique, monotonically decreasing sequence of numerical values. This result holds true for any sequence of standardly embedded torus knots *T*(2, 2*n* + 1) and torus links *T*(2, 2*n*). By this result we demonstrate that the computation of this adapted HOMFLYPT polynomial provides a powerful tool to measure topological complexity of various physical systems.

Vortex filaments in classical and quantum fluids^1^,^2^, magnetic flux tubes^3^, phase defects^4^, polymers and DNA macromolecules^5^,^6^ may interact and recombine through reconnection of neighboring strands. Details of the process depend on specific local mechanisms that may differ from case to case, but certain qualitative features — such as the preservation of the original strand orientation after recombination — are generic and common to all systems. In the majority of cases orientation-preserving reconnections occur when neighboring strands tend to align (at the time of closest approach) in an anti-parallel fashion^7^,^8^,^9^,^10^, before transversal merging and final separation. The prototype process is schematically shown in Fig. 1a, where the reconnection of two anti-parallel strands of a single structure (stage (i), in orange) produces a new structure given by a pair of loops (stage (ii), in orange and blue). In general, when two disjoint, closed tubes (representing vortex rings or DNA macromolecules) reconnect, the result is a single closed tube, and when a single closed tube reconnects with itself, the result is two closed tubes. Of course this process is not unique and depends crucially on the particular initial condition, as shown in Fig. 1b, where a trefoil vortex knot undergoing two simultaneous reconnection events produces two unlinked, unknotted vortex loops. The study of these processes is clearly of great importance, because the change of topology is often accompanied by a change in energy, entropy and function.

According to recent observations based on direct numerical simulations of decaying Bose-Einstein condensates^11^,^12^ and recombinant DNA plasmids^13^, both systems initially given by torus knots and links are found to undergo an almost identical cascade process through an alternate sequence of *T*(2, 2*n* + 1) torus knots and *T*(2, 2*n*) torus links as *n* (integer) decreases to 0. After every reconnection the knot/link seems to gradually untie by removing a single crossing at a time, by reducing consistently topological complexity (measured by the minimum number of crossings). Remarkably, this sequence of topological transitions seem to follow an identical topological decay pattern (shown in Fig. 2), irrespective of the physical context considered. This, we believe, is probably due to the particular choice of initial conditions given by the ideal shape configuration. Torus knots and links in ideal shape are maximally symmetric objects with optimal geometric regularity, crucial in the follow-up of the reconnection process. The counter-example of Fig. 1b (see also the envisaged process discussed in the section Concluding remarks) clearly shows how a very different initial condition far from ideal may produce a very different scenario.

In our recent derivation of Jones and HOMFLYPT polynomials for fluid knots^14^,^15^ we showed that these adapted polynomials can be used not only to detect topological differences between knots and links (as standard knot polynomials typically do), but also to *measure* topological differences between physical states. Here, by using our adapted HOMFLYPT polynomial we show that the particular cascade above can be detected by a unique, monotonically decreasing sequence of numerical values.

## Methods

In general, polynomial computations for knots and links are based on the recursive application of two *skein relations*, by the use of diagrams^16^ obtained from the indented projection of a given knot with minimal crossings (see top diagram of Fig. 3). For tubular filaments these diagrams simply represent the tube centerline, with over-crossings and under-crossings to denote respectively over-passes and under-passes of tube strands. The first skein relation simply states that the polynomial value of the unknot (the trivial knot) is equal to 1. The second skein relation prescribes how a “state” associated with a particular crossing type, represented for example by the over-crossing 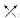, is related to its opposite, the under-crossing 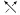, and the relative “smoothing” given by two parallel strands (the non-crossing 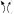). Standard knot polynomials are powerful topological invariants readily available from dedicated web-sites (such as *KnotAtlas*^17^). By identifying a physical knot with a space curve *K* of physical strength *Φ* (circulation, in the case of a vortex), we can extend standard knot polynomials by new polynomials that characterize physical knots in terms of knot type and strength^15^,^18^.

For physical knots the skein relations of the HOMFLYPT polynomial *P*_*K*_(*a, z*) (a two-variable polynomial in *a* and *z*) are given by^15^


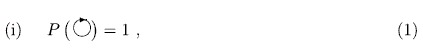



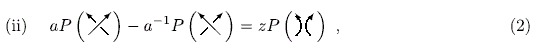


with *z* = *k* − *k*^−1^ and


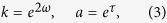


where *ω* = *λ*_*ω*_*Wr* and *τ* = *λ*_*τ*_*Tw* denote respectively reference values of writhe *Wr* and twist *Tw* of *K*^19^,^20^, weighted by the relative uncertainty (probability) factors {*λ*_*ω*_, *λ*_*τ*_} ∈ (0, 1).

Since *a* and *z* admits respectively interpretation in terms of twist and writhe (independently), eq. (2) prescribes a relationship between twist contribution (from the left-hand-side) in terms of writhe. Hence eq. (2) provides a framing prescription for physical knots: thus, reduction of HOMFLYPT polynomial to Jones, that is a one-variable polynomial, implies that


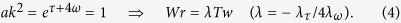


## Results

Let us make the following assumptions:

1. all torus knots *T*(2, 2*n* + 1) and links *T*(2, 2*n*) (with *n* = 0, 1, 2, …) considered here are standardly embedded on a mathematical torus in closed braid form;
2. all torus knots *T*(2, 2*n* + 1) and links *T*(2, 2*n*) form an ordered set {*T*(2, *n*)} (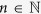) of elements listed according to their decreasing value of topological complexity given by the minimum number of crossings *c*_min_ = *n*;
3. any topological transition between two contiguous elements of {*T*(2, *n*)} is determined by a single, anti-parallel reconnection event^21^.

We can then prove the following, general result.

**Theorem.** *Let us consider the family* {*T*(2, *n*)} *of torus knots and links. The HOMFLYPT polynomial of T*(2, *n*) *is given by*


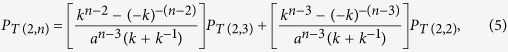


where *P*_*T*(2,3)_ = 2*a*^−2^ + *a*^−2^*z*^2^ − *a*^−4^ and *P*_*T*(2,2)_ = *a*^−1^*z* + (*a*^−1^ − *a*^−3^)*z*^−1^.

*Proof.* Let us consider the diagram of *T*(2, *n* + 2), and in particular a portion of it represented by the top diagram of Fig. 3 and apply the second skein relation to relate crossing sites. From (2) we have


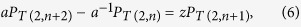


that is


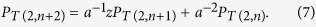


This is a recurrence relation between polynomials, so it can be written as


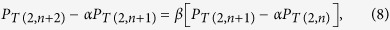


where *α* and *β* are two undetermined coefficients. By comparing (8) and (7) we have *α* + *β* = *a*^−1^*z* and *αβ* = −*a*^−2^, that combined together give a quadratic equation, with solutions


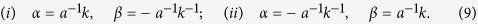


On the other hand, by applying (8) recursively we have


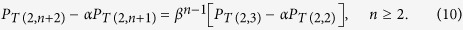


By rewriting (10) and computing recursively each contribution, we have


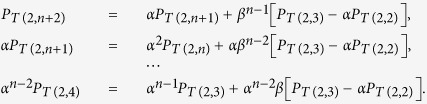


Now, by substituting consecutively each term we have


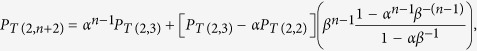


that is


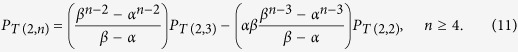


By direct substitution we can see that both solutions (9) reduce (11) to the same, identical recurrence equation given by (5). This completes the proof. ◼

We can now apply the specifications (3). For simplicity, and without loss of generality, let’s set *Φ* = 1. By using (3) we can re-write (5) as


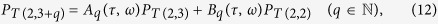


where


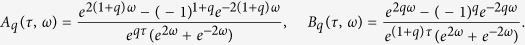


**Figure 2:**
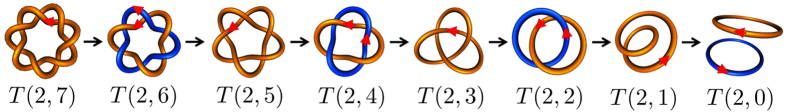
Cascade of torus knots and links produced by single reconnection events. Time sequence of a cascade of torus knots (orange online) and links (blue and orange online) produced by single reconnection events, that consistently reduce topological complexity by stepwise unlinking: in the example the torus knot *T*(2, 7) is shown to gradually decay to a pair of unknotted, unlinked loops *T*(2, 0) through the alternate production of distinct torus knots and links.

**Figure 3:**
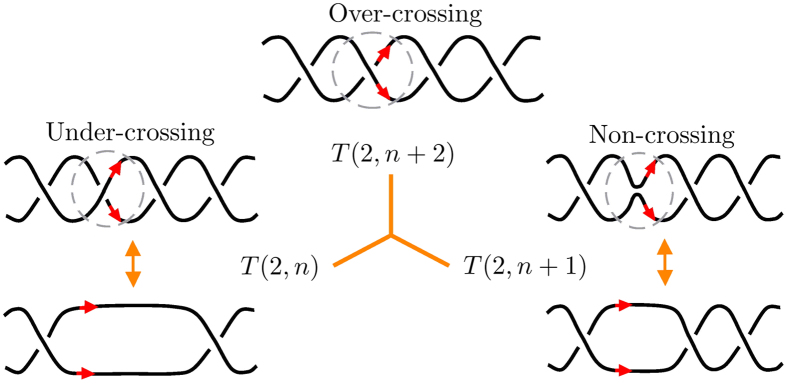
Application of the skein relation (2) to a portion of torus knot/link diagram. The skein relation (2) prescribes a relationship between the polynomial of the knot represented by the top diagram (with over-crossing encircled) and the polynomials of the knots represented by the bottom diagrams (with under-crossing and non-crossing sites encircled). Double arrows denote topological equivalence between diagrams. By direct application of eq. (2) to the top diagram we can determine the corresponding relationship between *P*_*T*(2, *n*+2)_, *P*_*T*(2,*n*)_ and *P*_*T*(2,*n*+1)_.

The family of torus knots and links considered earlier^11^,^12^,^13^ correspond to *positive T*(2, *n*) (i.e. of type shown in Fig. 2, with all over-crossings); for this knot family, denoted by {*T*(2, *n*)}_+_ reference values for *Wr* and *Tw* must be taken between 0 and 1^22^. For the family {*T*(2, *n*)}_−_ of mirror knots with all under-crossings, i.e. for *negative T*(2, *n*), the HOMFLYPT polynomial can be readily obtained by using the following lemma.

**Lemma.** *Given the knot K, the HOMFLYPT polynomial of the mirror knot* 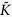 *is given by replacing*


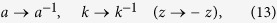


*into the HOMFLYPT polynomial of the knot K*.

A straightforward proof of this Lemma is obtained by using the second skein relation (2).

For negative *T*(2, *n*) reference values for *Wr* and *Tw* must be taken between −1 and 0^22^. Note that by using (13) and by replacing *τ* → −*τ* and *ω* → −*ω*, equation (12) remains unchanged. Hence, for *any* choice of *Wr* and *Tw* (consistently with positive or negative *T*(2, *n*)) and for *any* choice of the uncertainty values {*λ*_*ω*_, *λ*_*τ*_} ∈ (0, 1) we obtain, for decreasing *n*, a monotonically decreasing sequence of HOMFLYPT values. We can thus prove the following corollary in full generality.

**Corollary.** *Let us consider the family of torus knots and links* {*T*(2, *n*)}_±_*. HOMFLYPT computation of P*_*T*(2,*n*)_ *generates, for decreasing n (*
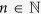
*), a monotonically decreasing sequence of numerical values given by (12).*

**Figure 4:**
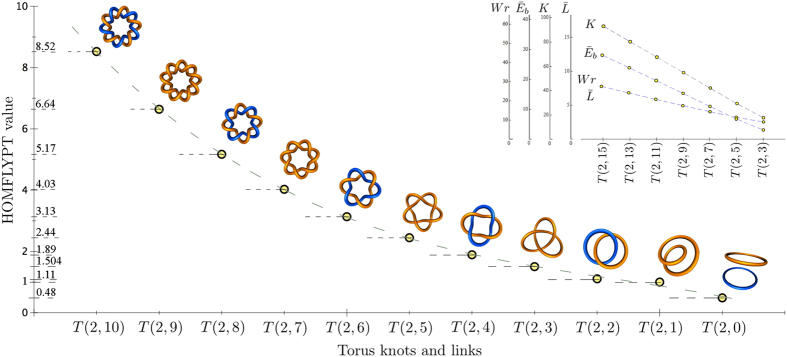
Monotonically decreasing sequence of values obtained by HOMFLYPT computation. Inset: generic behavior of global geometric properties of positive torus knots. HOMFLYPT computation given by (12) for positive *T*(2, *n*), with *λ*_*ω*_ = *λ*_*τ*_ = 1/2, |*Wr*| = |*Tw*| = 1/2 and *n* = 4, 5, …, 10: for decreasing *n* we obtain a monotonically decreasing sequence of numerical values (circles denote exact values, the dashed curve represents the best fit given by a quartic polynomial). Inset: typical behavior of global geometric properties of positive torus knots: writhe *Wr*, total squared curvature 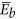, total curvature *K* and total length 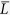 (over-bars denote non-dimensional quantities)^22^.

Initial values are given by the HOMFLYPT computation of the 2-component unlink unknot *T*(2, 0), unknot *T*(2, 1), Hopf link *T*(2, 2) and trefoil knot *T*(2, 3)^15^. For the sake of example we can take *λ*_*ω*_ = *λ*_*τ*_ = 1/2 (equivalent to an equi-distribution of uncertainty of writhe and twist values) and |*Wr*| = |*Tw*| = 1/2 to obtain the numerical values of the sequence shown in Fig. 4. Other values of *λ*_*ω*_ and *λ*_*τ*_ would equally give a monotonically decreasing sequence.

The sequence is clearly independent from the specific physical context and it appears to be consistent with the typical behavior of global geometric properties of standard torus knots. Computations of various quantities, such as first-order elastic energy (bending and torsional) of thin rods^22^ and kinetic energy of vortex filaments^23^ (see inset of Fig. 4) demonstrate a remarkable similarity with the monotonically decreasing sequence of HOMFLYPT values.

## Concluding Remarks

**Figure 5:**
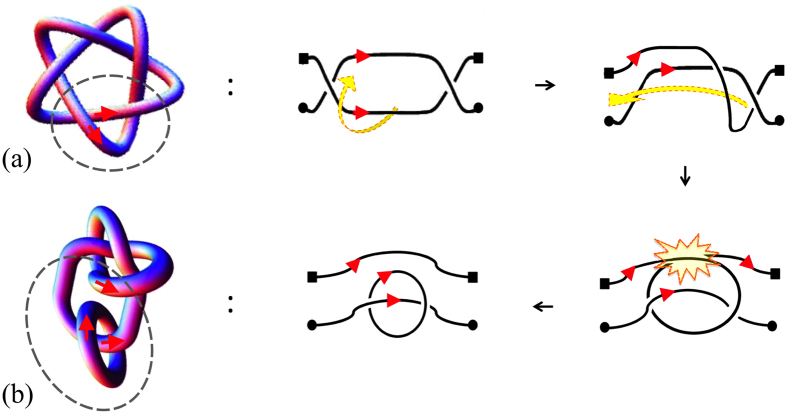
A strand of the standardly embedded knot *T*(2, 5) in the encircled region can deform and undergo a reconnection to form the connected sum of a trefoil knot and a Hopf link. (**a**) The encircled region of the standardly embedded knot *T*(2, 5) is represented by the diagram shown in the center (top row). If one of the strands is deformed continuously to coil around the other, a reconnection of neighboring parts can take place by forming a loop (central diagram, bottom row) linked to the rest. (**b**) A connected sum of a trefoil knot and a Hopf link, denoted by 3_1#2_2_1 (topologically different from the connected sum of the standardly embedded *T*(2, 3)#*T*(2, 2)) is thus formed.

The results presented here hold true for any sub-sequence of standardly embedded torus knots and links in the family {*T*(2, *n*)}_±_ under the assumption that any topological transition between contiguous knot/link types in {*T*(2, *n*)}_±_ is produced by a single, anti-parallel reconnection event. Standard embedding implies maximal symmetry and minimal crossing presentation, that is, in a way, reminiscent of the ideal shape concept. Such a requirement mimics the initial conditions chosen for the cascade process^11^,^12^,^13^ and it is essential in the mathematical proof given above. A very different scenario can indeed be envisaged for an initial configuration far from “ideal”. Take for example the standardly embedded knot *T*(2, 5) shown in Fig. 5a. Consider the encircled region and its representation given by the central diagram (top row). Suppose to deform one of the strands continuously around the other (as indicated by the dashed arrows) so that two neighboring parts of the coiled strand reconnect to produce a single loop. The resulting system, shown in Fig. 5b, is the connected sum of a trefoil knot and a Hopf link denoted by 3_1#2_2_1 (topologically different from the connected sum of the standardly embedded *T*(2, 3)#*T*(2, 2)). This composite knot has same topological complexity (*c*_min_ = *n* = 5) of the original *T*(2, 5), but very different geometry and topology.

The same argument applied to the torus link *T*(2, 6) gives the composite knot 3_1#3_1 (connected sum of two trefoil knots), again with same topological complexity of *T*(2, 6) but very different geometry and topology. By using a basic property of the HOMFLYPT polynomial^24^ for composite knots (say *K*_1_ and *K*_2_), given by


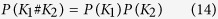


we can compute HOMFLYPT for the two cases above and compare these values with the values obtained for the sequence of standardly embedded knot types. We find


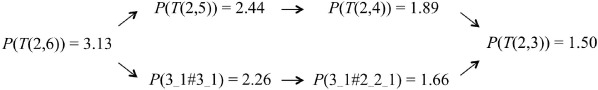


that shows that an alternative path of monotonically decreasing values (given by the bottom branch above) is possible if we drop the assumption (A1) of standard embedding. The same can happen for other knot types of higher topological complexity. Work to establish probability paths associated with alternative topological cascade scenarios is currently under way (Mariel Vasquez, private communication) and no doubt these results will help to choose more appropriate values for the probability factors *λ*_*ω*_ and *λ*_*τ*_.

What HOMFLYPT computational values may say in terms of physics is not yet clear. However, as shown by the inset of Fig. 4, there is an unambiguous correspondence between HOMFLYPT computations and various properties of physical knots. As discussed in earlier work^15^ the present results demonstrate that HOMFLYPT values — rooted in writhe and twist helicity contributions — provide new information to identify and *quantify* the topology of complex systems. If standard knot polynomials can help to classify different topologies, our adapted HOMFLYPT polynomial can measure these topological differences by attaching a number to any stage of the evolution. How these numbers can be related to energy (or some other fundamental physical property of the system) is not yet clear, but there is no question that further application of this technique to experimental or observational data will help to uncover and possibly establish new relationships between behavior of physical systems, energy and structural complexity. We believe this will open the door to a myriad of possible applications, from energy estimates in braided magnetic fields in solar flares to energy transfers in turbulent flows, from entropy measures in disordered systems to functional prescriptions of recombinant DNA plasmids.

## Additional Information

**How to cite this article**: Liu, X. and Ricca, R. L. Knots cascade detected by a monotonically decreasing sequence of values. *Sci. Rep.* **6**, 24118; doi: 10.1038/srep24118 (2016).

## Figures and Tables

**Figure 1:**
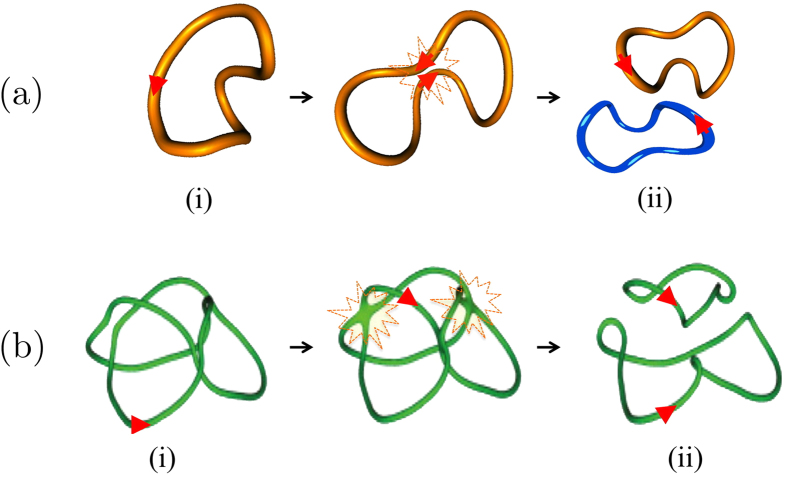
Time sequences of interaction and anti-parallel reconnection of (**a**) a physical filament in space and (**b**) a trefoil vortex knot governed by the Gross-Pitaevskii equation. Physical knots and links are identified by oriented tubular filaments (orientation denoted by arrows, red online) of given strength *Φ*. (**a**) Time evolution of (i) an unknotted, folded loop (orange online); when neighboring strands come into close contact in an anti-parallel fashion a reconnection event takes place by transversal merging. As a result (ii) a pair of unknotted, unlinked loops (blue and orange online) are produced. (**b**) Numerical simulation of the evolution of (i) a trefoil vortex knot governed by the Gross-Pitaevskii equation (images adapted from ref. 25). Vortex strands reconnect simultaneously at two different locations to produce (ii) a pair of distinct, unknotted, unlinked loops.
